# Temperature-Tunable 2D Assembly of PTCDA on Fe-Intercalated TaS_2_

**DOI:** 10.1021/acsomega.5c03930

**Published:** 2025-07-22

**Authors:** Hou-Ju Chen, Yu-Hsun Chu, Po-Hsi Huang, Chia-Nung Kuo, Chin Shan Lue, Minn-Tsong Lin

**Affiliations:** † Department of Physics, 33561 National Taiwan University, Taipei 10617, Taiwan; ‡ Department of Physics, 34912 National Cheng Kung University, Tainan 70101, Taiwan; § Taiwan Consortium of Emergent Crystalline Materials (TCECM), National Science and Technology Council, Taipei 10601, Taiwan; ∥ Institute of Atomic and Molecular Sciences, Academia Sinica, Taipei 10617, Taiwan; ⊥ Research Center for Applied Sciences, Academia Sinica, Taipei 11529, Taiwan; ## Program on Key Materials, Academy of Innovative Semiconductor and Sustainable Manufacturing, National Cheng Kung University, Tainan 70101, Taiwan

## Abstract

By controlling the postannealing temperature, atomically flat supramolecular assemblies of perylene-3,4,9,10-tetracarboxylic dianhydride (PTCDA) with tunable structural and electronic properties were synthesized on an intercalated quasi-two-dimensional crystal, Fe_0.24_TaS_2_, and characterized using scanning tunneling microscopy and spectroscopy (STM/STS). At 100 °C, a coexistence of a herringbone PTCDA assembly with p-type-like HOMO–LUMO alignment and a pseudosquare Fe–PTCDA structure were observed on the surface. Upon further annealing at 150 °C, a new dominant Fe–PTCDA honeycomb–kagome framework with chirality emerged, exhibiting n-type-like HOMO–LUMO characteristics. This transition in the types of HOMO–LUMO alignments, along with spatial variations in Fermi level shifting, is attributed to charge transfer from Fe atoms. These findings highlight the potential for designing functional materials, engineering interfacial properties, and constructing exotic low-dimensional structures—such as kagome metal–organic frameworks (MOFs) and chiral nanostructures—through the controlled synthesis of organic heterostructures with emergent crystal phases.

## Graphical abstract

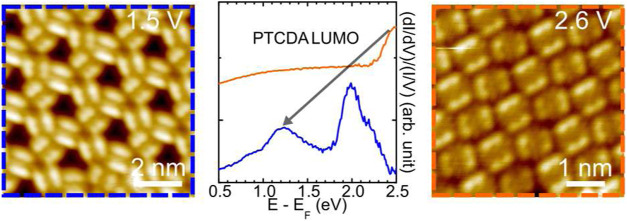

## 1 Introduction

Organic semiconductors, an important building block in modern technology, have encouraged the innovative designs of light-emitting diodes, solar cells, field-effect transistors, etc., benefiting applications in electronics, optoelectronics, and spintronics.^1,2^ The formation of ordered two-dimensional (2D) organic self-assemblies and frameworks expands the potential of organic heterostructures, in which tailored films and interfaces, as well as adjustable physical properties, are achievable via careful selection of materials, coordinating elements, and growth conditions.^3,4^

Perylene-3,4,9,10-tetracarboxylic dianhydride (PTCDA), a widely used electron acceptor in organic semiconductors, features conjugated pi-bonds, which allow for reasonable charge and spin transport.^5–8^ PTCDA forms 2D self-assemblies on noble metals, graphene, and transition metal dichalcogenides (TMDs), and generally behaves as an n-type semiconductor.^9,10^ Together with metal elements, PTCDA further establishes 2D metal–organic frameworks (MOFs). For example, deposited along with Fe, PTCDA forms ladder-like Fe-PTCDA networks after postannealing.^11^ In this ladder-like MOF, the PTCDA Fermi level depends on the number of coordinated Fe atoms, which shift the Fermi level to lower energy positions by charge transfer.^12–14^

As most 1D and 2D MOFs,^15–17^ the PTCDA MOFs reside on noble metal crystalline surfaces. However, no inherent constraints preclude their presence on other substrates, such as van der Waals (vdW) type crystals, which include many emergent materials with exotic physical properties.^18,19^ Through intercalations and surface decorations, organic modulations have been applied to vdW crystals ranging from 3D topological insulators to monolayer semiconductors.^20–24^ 2D MOFs prepared on vdW-based crystalline surfaces, nevertheless, remain relatively unexplored.^25,26^

In this work, we demonstrate the formation of 2D PTCDA-based structures on an intercalated vdW crystal: Fe_0.24_TaS_2_ at different postannealing temperatures. Fe-intercalated TaS_2_ crystals are layered metallic ferromagnets, in which an Fe interlayer bridges two 1H TaS_2_ layers.^27,28^ Fe intercalation introduces Fe-derived electronic states that modify the Fermi surface and shift the Fermi level relative to pristine 2H TaS_2_.^29^ These electronic changes suppress the charge density wave (CDW) instability characteristic of 2H-TaS_2_, resulting in behavior more typical of conventional metals.^30^ On the other hand, these crystals may stay ferromagnetic with thicknesses down to a few layers and notably survive ambient conditions for months, which makes them highly promising in low-dimensional spintronics.^31^ Using scanning tunneling microscopy (STM), we observed cleaved Fe_0.24_TaS_2_ surfaces with residual Fe adatoms from the disassembled Fe-interlayer, which form MOFs after the deposition of PTCDA and postannealing. By adjusting the annealing temperature, several atomically flat 2D PTCDA-based structures can form on the Fe_0.24_TaS_2_ surface, including self-assemblies, an Fe-PTCDA pseudosquare phase, and a distorted honeycomb-kagome MOF. Scanning tunneling spectroscopy (STS) of the 2D PTCDA structures reveals a switch in molecular doping types from the herringbone assembly with a p-type-like HOMO–LUMO alignment to the honeycomb-kagome MOF with a n-type-like HOMO–LUMO alignment. The variety of 2D PTCDA structures on Fe_0.24_TaS_2_ suggests potentially practical and exotic organic heterostructures with emergent crystals.

## 2 Results

In Fe_*x*_TaS_2_, the Fe intercalation occurs in the vdW gap between two 1H TaS_2_ layers, such as the stoichiometric Fe_0.25_TaS_2_ crystal illustrated in Figure 1a.^32^ Figure 1b,c are STM images of *in situ* cleaved F_0.24_TaS_2_ surfaces, on which flat domains hundreds-of-nanometer-wide exist and are decorated by atomic protrusions. To minimize the influence of cleavage-induced variability, we examined both sides of a cleaved crystal and observed that each surface was decorated with atomic-scale protrusions, as shown in Figure 1b, indicating consistent surface features across different cleavage planes. The zoomed-in image in Figure 1c reveals the surface’s periodic structures, indicated by two black triangles. The small triangle represents the surface S lattice, while the large one corresponds to a characteristic 2a × 2a superstructure in Fe_0.25_TaS_2_ due to an ordered arrangement of Fe ions in the van der Waals gaps, along with associated charge transfer effects.^30^ Figure 1d shows a height profile across the surface superstructures and protrusions, of which the heights are about 20 and 140 picometers, respectively and suggest that the protrusions are adatoms on the surface. Since the flakes were cleaved *in situ* in ultrahigh vacuum and directly transferred to the STM, the atomic protrusions most likely originate from the remaining Fe adatoms from disassembled Fe-intercalation layers rather than from surface contamination. Energy-dispersive X-ray spectroscopy (EDS) analysis (Figure S1) also confirms the absence of extraneous elements, and the elemental maps show a homogeneous distribution of Fe, Ta, and S on the micrometer scale, ruling out surface contamination. The STM results also unveil that the Fe adatoms prefer to sit on the S top-site, as indicated by the crossing point of three black lines, which represent the S atom arrays in Figure 1c.

**Figure 1:**
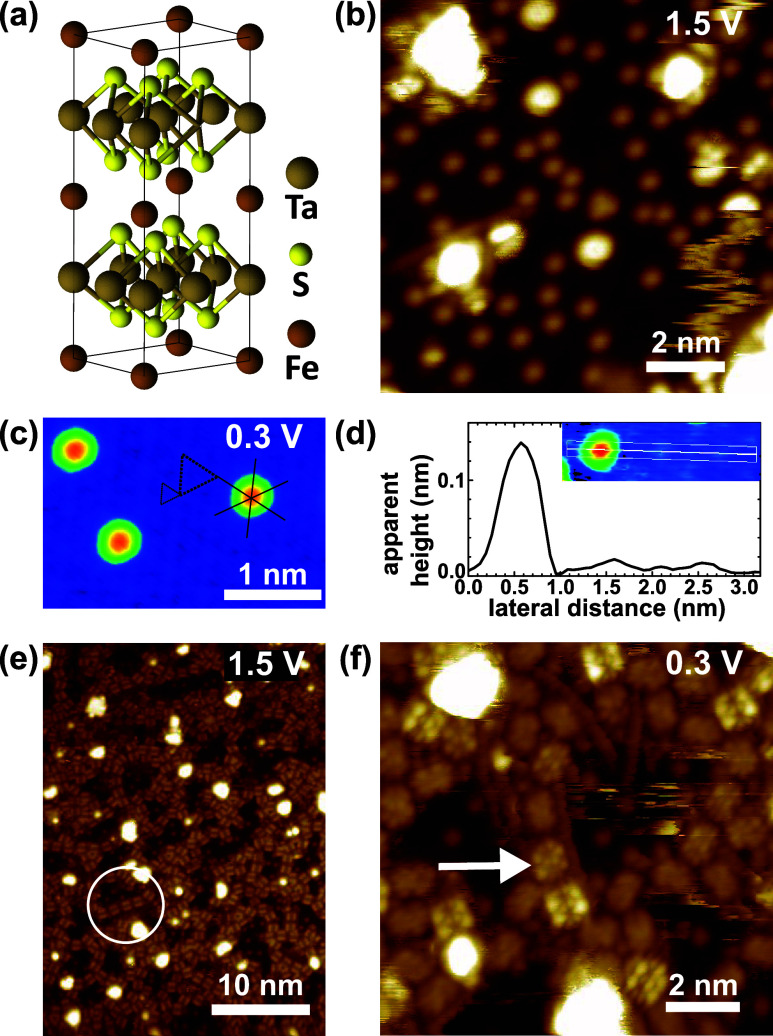
(a) Crystalline structure illustration of Fe_0.25_TaS_2_. STM images of *in situ* cleaved Fe_0.24_TaS_2_ are shown in (b) and (c). Two black dashed triangles in (c) mark the surface S lattice and the 2 × 2 superstructure, and three black straight lines represent the S atom alignments. (d) An apparent height profile across the surface protrusion and superstructure corrugations in the inset image. (e) and (f) STM images of Fe_0.24_TaS_2_ after deposition of submonolayer PTCDA. An Fe-PTCDA chain is circled in white in (e), and a molecule with the LUMO appearance is marked by a white arrow in (f).

Figure 1e showcases an STM image of Fe_0.24_TaS_2_ surfaces decorated by a submonolayer of PTCDA molecules before postannealing. The molecules exhibit a double-lobe feature, which is the result of the lowest unoccupied molecular orbital (LUMO) probed at a large tip-molecule distance with bias voltages higher than the LUMO energy.^33^ Among the disordered molecule distribution, there exist 1D PTCDA chains (circled in white). The PTCDA chain is a typical 1D metal-PTCDA framework on Au(111).^11,34^ In these 1D chains, every two PTCDA molecules are bridged by two Fe atoms via a pair of O–Fe–O bonds. The existence of PTCDA chains agrees with the idea of residual Fe adatoms on cleaved Fe_0.24_TaS_2_ surfaces. A zoomed-in image at 0.3 V in Figure 1f shows that molecules within the same Fe-PTCDA chain may have different appearances at the same energy. The PTCDA molecule pointed by a white arrow exhibits the characteristic LUMO pattern,^33^ while the one below it possesses double lobes, and the one above it shows no LUMO features. The inconsistent molecule appearances at the same bias voltage suggest discrepancies in the Fermi level shifting due to locally modified charge transfer by additional Fe adatom or adsorbate attachments.

Targeting organized frameworks, we annealed the PTCDA-decorated Fe_0.24_TaS_2_ at 100 °C for 20 min. This treatment led to Fe aggregation as well as assembled and reorganized PTCDA, corresponding to the higher and lower islands in Figure 2a, respectively. Importantly, STM topographies recorded away from the molecular islands reveal clean (inset of Figure 2a), atomically flat regions with minimal point defects. The observed surface periodicity and superstructure in Figure 2c match those in Figure 1c, indicating that the underlying substrate remains well-ordered and uniform after subsequent annealing. Regarding PTCDA molecules, two phases of 2D PTCDA structures can be identified from the PTCDA submonolayer. The first phase, shown in Figure 2b, is a typical herringbone structure composed of PTCDA molecules connected to each other via quadrupolar interactions and weak O ·· H–C bonds, and has been observed across various substrates.^35–37^ The second phase is a “pseudo-square” network comprising misaligned, square-like cells (white dashed squares in Figure 2c) without long-range periodicity. We adopted the naming due to the resemblance of the cells to the known square PTCDA self-assembly and Fe-PTCDA framework.^11,38^ PTCDA in the herringbone assembly in Figure 2b exhibits a uniform LUMO double-lobe at 2.6 V, suggesting comparable LUMO energies of the molecules. The dI/dV spectrum of the PTCDA herringbone phase (Figure S3) shows a HOMO energy peak at −1.0 V and a LUMO energy peak at 2.7 V, giving a HOMO–LUMO interval of 3.7 eV, close to typical values for physisorbed submonolayer PTCDA.^10^ On the contrary, molecules in the pseudosquare phase could exhibit different appearances, as shown in Figure 2c, in which some of the molecules show clearer LUMO features compared to the others at 1.5 V. The lower LUMO energy and the spatial variation of MO energies suggest the involvement of Fe with nonuniform connections to the molecules, in which the Fe charge transfer shifts the LUMO toward the Fermi level. A pseudosquare structure with consistent molecular appearance and a higher LUMO energy could also be found (inset in Figure 2c), which could be a pure PTCDA self-assembly with a similar structure but without Fe.

**Figure 2:**
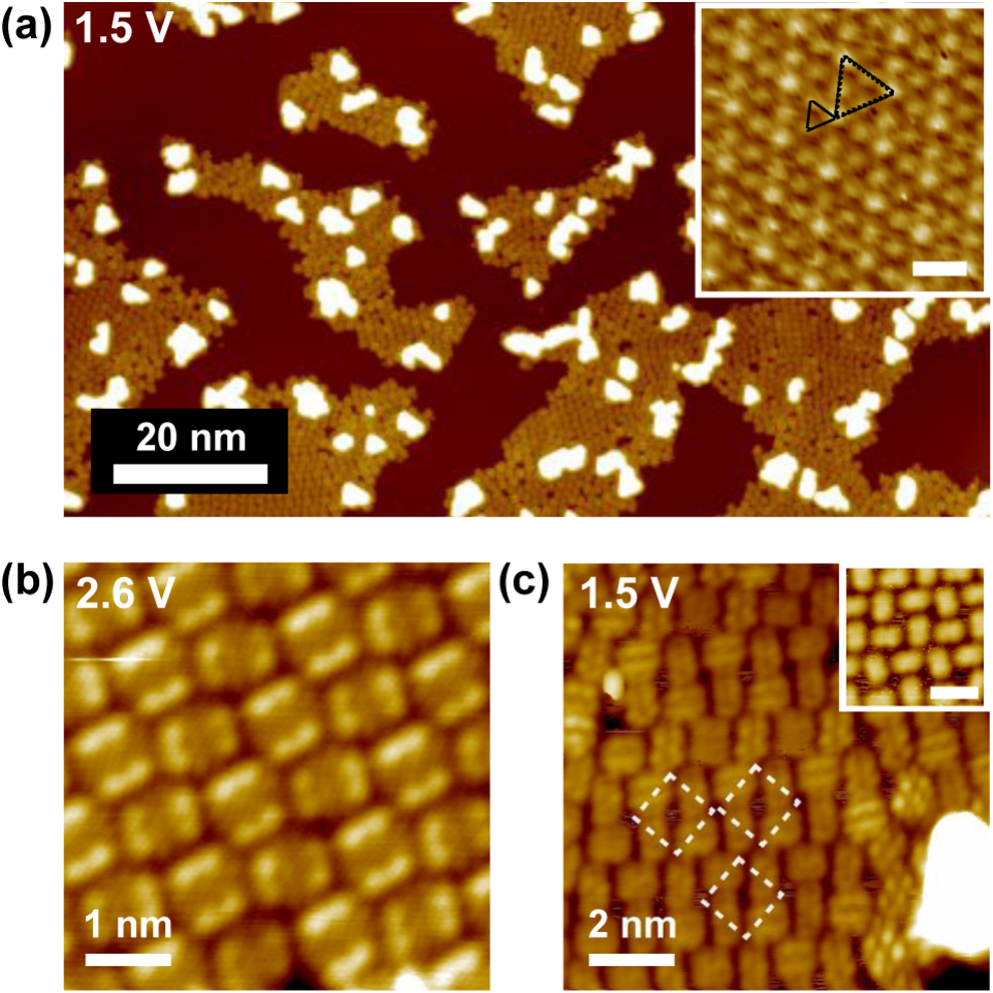
(a) An STM image of PTCDA covered Fe_0.24_TaS_2_ after postannealing at 100 °C. Inset: STM topographs with atomic resolution recorded away from the islands. The scale bar in the inset is 0.6 nm. Two main ordered structures are shown in the STM image in (b): herringbone PTCDA and (c): pseudosqaure Fe-PTCDA structures. White dashed squares in (c) mark the pseudosquare cells. Inset: pseudosquare structure with uniform molecular appearance at 2 V. The scale bar in the inset is 1.5 nm.

Further annealing at 150 °C for another 20 min induced a single and ordered honeycomb-shape PTCDA network along with Fe nanoislands, as shown in Figure 3a. A fast Fourier transform image of a larger-scale STM image is shown in Figure 3b and unveils two sets of 6-fold symmetric points, which correspond to the two preferred orientations of the networks, marked by white dashed lines in Figure 3a. A zoomed-in STM image in Figure 3c presents PTCDA with the double-lobe feature of the network at 1.5 V, while the bottom third of the image was scanned at 0.8 V for mapping the 2a × 2a superstructure and the surface S-lattice. Extending the S-lattice (white diamond mesh) to the PTCDA region, we constructed a ball–stick model of the PTCDA network on top of a theoretical S-lattice of the Fe_0.25_TaS_2_ (Figure 3d), in which the PTCDA placement was estimated based on the double-lobe feature.^32,39^ The unit cell, represented by an orange parallelogram, has a side length of 2.62 nm, agreeing with the experimental periodicity of about 2.5 nm.

**Figure 3:**
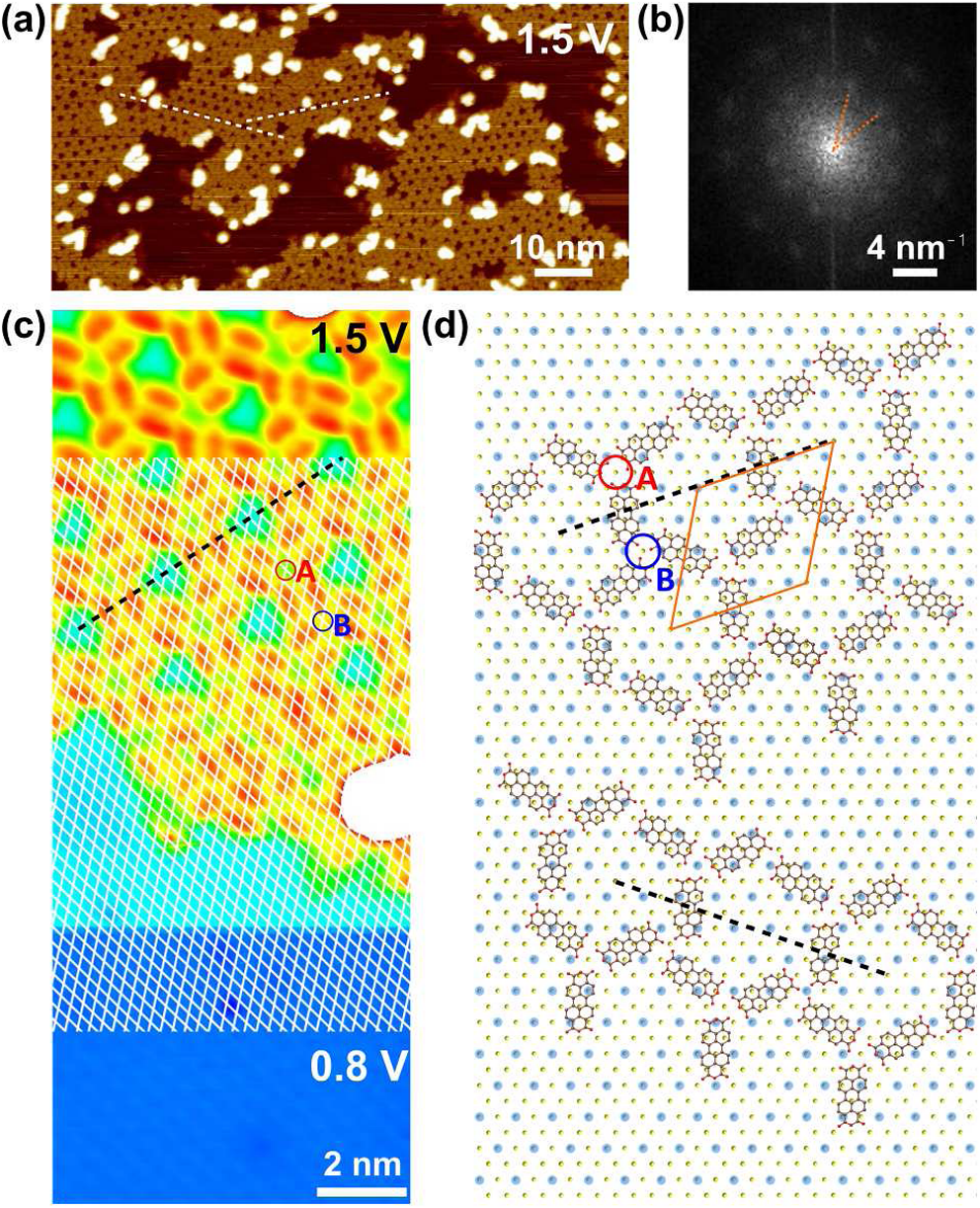
(a) An STM image of the distorted Fe-PTCDA honeycomb-kagome MOF, and (b) the corresponding FFT image. Two white dashed lines in (a) indicate two preferred orientations of the MOF. The angle between the orientations, represented by the orange dashed lines in (b), is around 37.5°. (c) An STM image mapped at 0.8 V (bottom) and 1.5 V (top). The surface S lattice extracted from the bottom part is presented by a white diamond mesh and extended to the top part. An accordingly constructed ball–stick model of the MOF is presented in (d). The yellow, brown, and red atoms represent S, C, and O atoms, respectively. The 2a × 2a superstructure is represented by pale blue dots. The MOF orientations are marked by black dashed lines, and two types of kagome triangular corners are marked A and B in both (c) and (d). The orange parallelogram in (d) represents the unit cell of the MOF. (Fe atoms are not shown in this ball-and-stick model.).

From the ball–stick model, it is clear that the PTCDA arrangement is a distorted kagome lattice with two unit triangles labeled A and B, of which the orientations are opposite. The O atoms on the short side of PTCDA surround the center of the triangles and are closer to each other at the triangle B compared with the triangle A case. This O–O connection between the molecules hardly agrees with the formation mechanism of PTCDA self-assemblies, which are mainly quadrupolar interactions and hydrogen bonds, and suggests the existence of Fe atoms at the triangle centers, bridging the molecules and forming an Fe-PTCDA MOF. Both triangles center at the surface S top-site, which is the preferred absorption site of Fe adatoms according to the STM observation in Figure 1c. Besides, X-ray photoelectron spectroscopy (XPS) analysis (Figure S2) shows a Fe 2p peak at 710.7 eV and an O 1s peak at 530.3 eV, indicating Fe–O and C–O–Fe bonding, respectively—providing evidence of Fe–PTCDA coordination consistent with metal–organic framework formation. The involvement of Fe is also supported by the LUMO energy, which is lower than the PTCDA herringbone self-assembly case presumably due to Fe charge transfer. In this case, the Fe atoms can be viewed as an additional honeycomb lattice, and the Fe-PTCDA MOF turns out to be a distorted honeycomb-kagome MOF. The distortion is caused by the restricted honeycomb lattice by the top-site adsorption of Fe, forcing the PTCDA molecules to tilt. It should be noted that the 2a × 2a superstructure (pale blue dots in Figure 3d) does not correlate with the kagome triangles and, therefore, growths of similar MOFs are possible for Fe-intercalated TaS_2_ with other Fe ratios.

Figure 3d also indicates that the molecular arrangement in the unit triangles shows no space inversion symmetry and exhibits chirality. Defining the PTCDA arrangement in the top half of Figure 3d as clockwise, we can construct a counterclockwise counterpart as in the bottom half, of which the MOF unit cell is accordingly rotated. As a result, there are two preferred orientations for the distorted honeycomb-kagome MOF, as the experimental observation. From the ball–stick model, the angle between the two MOF orientations is approximately 38°, consistent with the 37.5° angle between the two sets of 6-fold symmetry points in the FFT in Figure 3b. The broken inversion symmetry and the emergence of chiral motifs in the honeycomb–kagome Fe–PTCDA metal–organic framework (MOF) highlight its potential as a candidate for chiral nanostructures.^40^ Furthermore, the use of Fe-intercalated TaS_2_-a substrate with a well-defined hexagonal lattice—offers a promising platform for the integration of this MOF with other two-dimensional (2D) materials, enabling the construction of heterostructures with tailored electronic and structural functionalities.

Energy-dependent STM images of the honeycomb-kagome MOF are presented in Figure 4. While the double-lobe PTCDA is observed at 1.5 V (Figure 4a), clearer LUMO patterns appear at 1 V (Figure 4b). STS measurements were conducted to better unveil the electronic states of the framework, from which a peak at around 1.3 V appears in the STS curve in Figure 4d. Compared with the STS curve from the Fe_0.24_TaS_2_ substrate, which does not contain such peaks in the same energy range, detection of the substrate state through the molecules can be excluded. Combined with the LUMO observation in Figure 4a,b, the peak is reasonably attributed to the PTCDA LUMO. Another peak at around −0.8 V in the molecule STS curves in [Fig fig4](d), however, overlaps with a strong substrate state and, therefore, cannot be attributed to the highest occupied molecular orbital (HOMO) directly. The STM image, on the other hand, shows the PTCDA HOMO at −2.1 V (Figure 4c). In STS, since the HOMO peak seemed buried by substrate states or exponential backgrounds, we applied normalized STS: (d*I*/d*V*)/(*I*/*V*) to emphasize the molecular orbital peaks.^41^ Figure 4e presents the original and normalized STS curves from the honeycomb-kagome PTCDA in the HOMO energy range, and a peak is found at around −2.2 V, agreeing with the STM observation in Figure 4c. The HOMO–LUMO gap in the honeycomb-kagome framework is, therefore, approximately 3.5 V, close to typical values for physisorbed submonolayer PTCDA.^10,42^ It is worth noticing that the LDOS remains finite between −0.5 and 1.0 eV, likely due to contributions from the metallic Fe_0.24_TaS_2_ substrate. This behavior is reminiscent of previous reports on PTCDA herringbone monolayers on Au(111), where the metallic character of the substrate significantly influenced the apparent LDOS of the molecular layer.^43^

**Figure 4:**
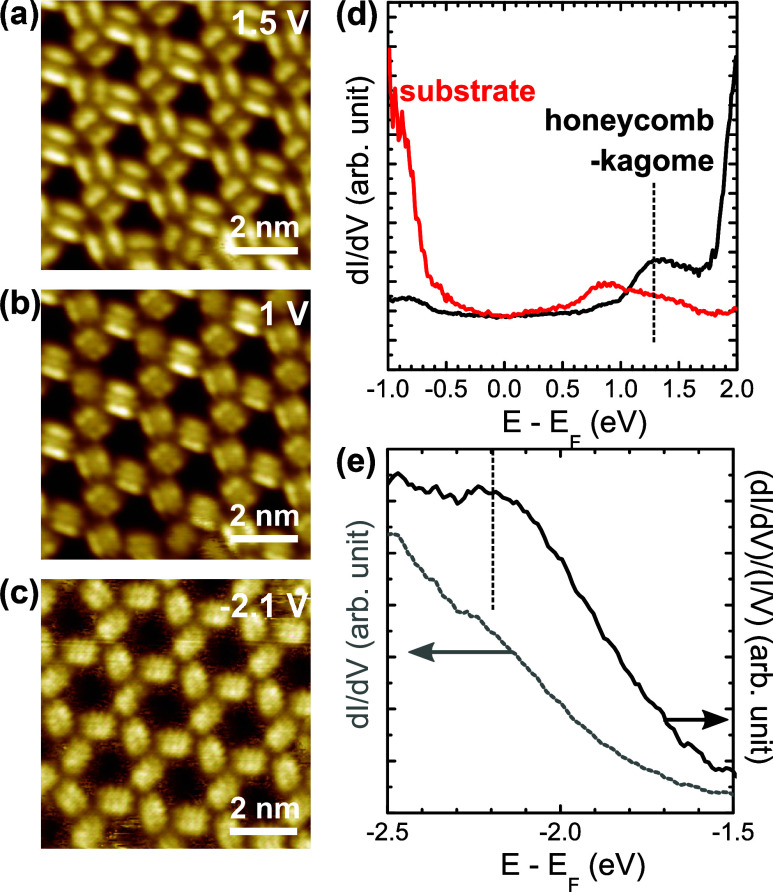
STM images of the honeycomb-kagome MOF taken at 1.5 V (a), 1 V (b), and −2.1 V (c). STS curves of the molecules in the honeycomb-kagome MOF are plotted in (d) and (e). An STS curve taken on the Fe_0.24_TaS_2_ surface is compared with the PTCDA case in (d), in which the PTCDA LUMO peak is indicated by a black dashed line. The PTCDA STS in the negative bias range is shown in (e), and normalized STS is performed to emphasize the HOMO peak marked by a dashed line.

## 3 Discussion

To compare the electronic properties among the aforementioned 2D PTCDA structures, a collection of corresponding STS curves is presented in Figure 5a. LUMO peaks in the curves are determined via comparisons to the appearing energies of the LUMO in STM images and indicated by colored dashed lines. Normalized STS were carried out to accentuate the LUMO peaks, and STM images of the corresponding molecules, from which the curves were taken, are presented next to the graph as Figure 5b,c. Figure 5b shows an edge region of the Fe-PTCDA distorted honeycomb-kagome MOF. The edge molecules circled in red are one-end open and, compared to their fully coordinated counterparts in the bulk region, connect to only half the Fe atoms. Assuming there is one Fe atom at each honeycomb lattice point, each bulk PTCDA molecule effectively connects to two-thirds of Fe atoms, while the edge PTCDA connects to one-third of Fe atoms. STS curves of these two cases, presented in Figure 5a, reveal an LUMO approximately 0.3 eV higher in the PTCDA edge, which is in agreement with the predicted lower Fe charge transfer to the edge molecules.^12,44^

**Figure 5:**
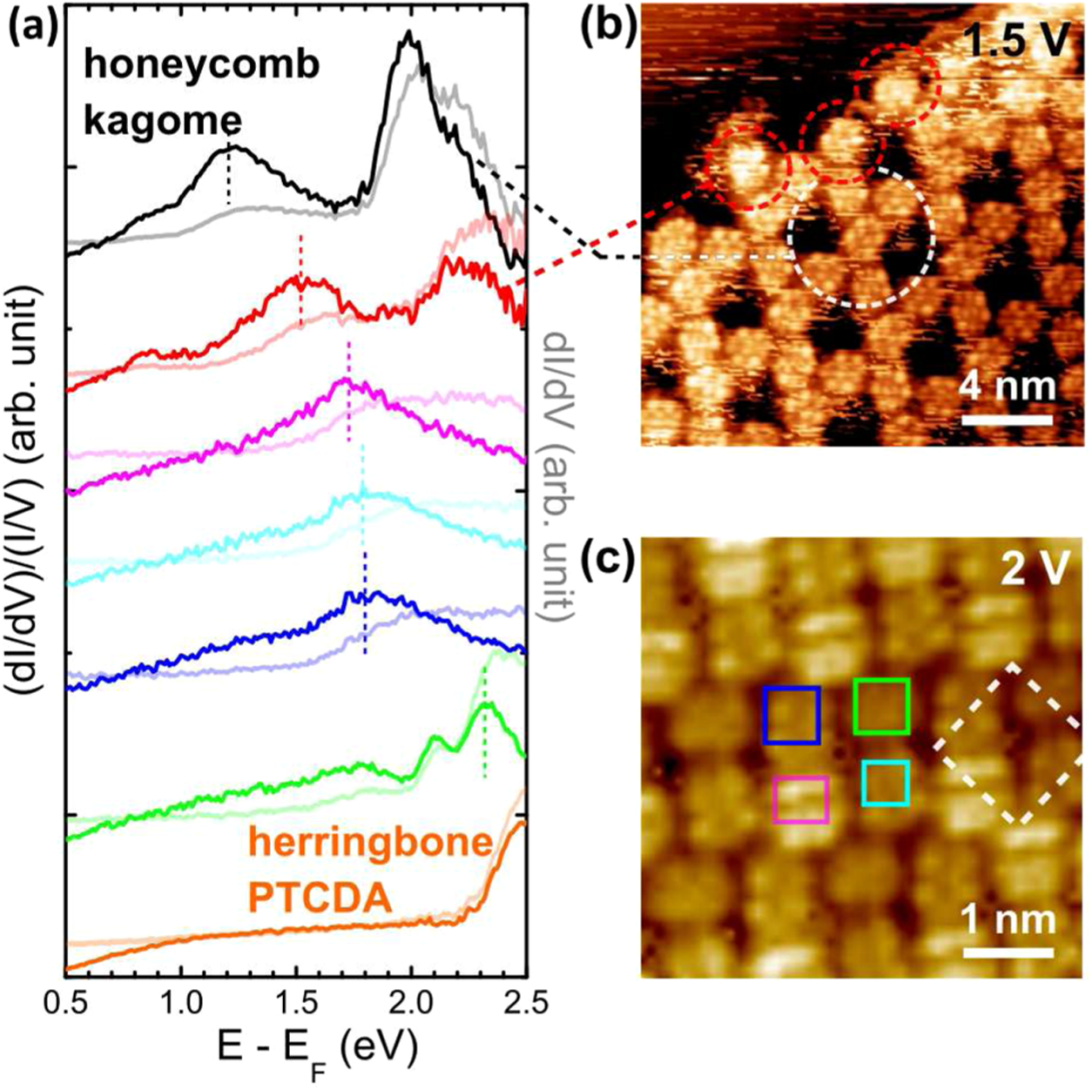
(a) STS and the normalized curves taken from the PTCDA assembly and MOFs. The original dI/dV curves are presented in pale colors. (b) An STM image of the PTCDA honeycomb-kagome MOF. Edge and bulk PTCDA molecules, from which the corresponding STS curves in (a) were taken, are circled in red and white. (c) An STM image of the pseudosquare phase Fe-PTCDA, of which one of the molecules (marked by a green square) in the square cell does not show LUMO as the others. The rectangles mark the region where the STS curves in (a) with the same colors were taken. The white dashed square marks the cell.

As shown in Figure S3, the HOMO and LUMO positions of the herringbone phase are located at −1.0 and +2.7 eV, respectively, indicating a relatively rare behavior that the Fermi level lies closer to the HOMO. We therefore refer to this as a “p-type-like HOMO–LUMO alignment.” In contrast, for the Fe-coordinated honeycomb–kagome phase, STS reveals HOMO and LUMO features at approximately −2.2 and +1.3 eV, respectively, with the Fermi level situated closer to the LUMO. This is referred to as an “n-type-like HOMO–LUMO alignment.” PTCDA often acts as an acceptor in organic semiconductors due to high electron affinity and exhibits n-type-like HOMO–LUMO energy interval on metallic substrates for substrate image charge, interface gap states, charge transfer, etc.^9^ The existence of a PTCDA layer with p-type-like HOMO–LUMO alignment could rely on weak interaction with the substrate and be a useful component of the multilayer heterostructures. Comparing the STS curves taken from the PTCDA-based 2D structures in Figure 5a, we observe a switch of the PTCDA HOMO–LUMO alignment type from p-type-like to n-type-like when there is enough charge transfer from the coordinating Fe atoms.

Molecules in the pseudosquare phase, on the other hand, exhibit LUMO energies in between the herringbone and the kagome cases in Figure 5a. STS curves from the four molecules inside the square-like cell in Figure 5c were averaged over the regions marked by colored rectangles. The LUMO energies of the blue, cyan, and pink molecules are about 1.7 to 1.8 eV, approximately 0.5 eV lower than that of the green molecule, which corroborates the STM observation that only the green molecule does not show the LUMO pattern at 2 V in Figure 5c while the other three do. Being higher than the other molecules, the pink molecule has similar STS features to the blue and cyan molecules. This indicates that the topographic variations do not lead to different LUMO shifts and the discrepant LUMO energies more likely result from uneven Fe connections to the molecules within the square-like cell.

Moreover, our discovery of these three distinct PTCDA assemblies with tunable HOMO–LUMO alignment types and electron doping intensity on the same substrate enables control over interfacial electronic properties. Such tunability is critical for engineering band alignment in heterostructures used in organic semiconductors, photodetectors, and sensors.^45–47^ Our results therefore provide a valuable foundation for future studies that aim to couple tunable organic frameworks with 2D semiconductors in functional nanoelectronic devices.

It is noteworthy that both the edge and bulk PTCDA molecules in the honeycomb framework exhibit a second prominent peak of about 0.8 V higher than the LUMO peak in STS. This peak holds several potential origins, including LUMO+1, LUMO splitting, and flat bands associated with the honeycomb-kagome structure. The energy gap between LUMO and LUMO+1 is significantly larger than 0.8 eV, ruling out that possibility. On the other hand, LUMO splitting would typically result in a smaller HOMO–LUMO gap due to the lowered LUMO energy.^13,48,49^ However, STS curves in Figure 4 do not exhibit this narrowing. Since kagome band features seem absent within the HOMO–LUMO gap of honeycomb-kagome PTCDA, a flat band away from the Fermi level could be an alternative explanation to the observed peak.^26,50,51^ Solving the exact nature of this peak will require further investigation through detailed STS measurements and ab initio calculations.

## 4 Conclusions

In summary, our work demonstrates the successful synthesis of PTCDA assemblies and MOFs on the Fe_0.24_TaS_2_ surface. We resolved the molecular arrangement in the distorted Fe-PTCDA honeycomb-kagome framework and highlighted its n-type-like HOMO–LUMO alignment, driven by charge transfer from Fe atoms. This contrasts with the herringbone phase with p-type-like HOMO–LUMO alignment and emphasizes the tunability of charge-transfer-induced electron doping within these structures. The presence of an intermediate pseudosquare phase and local variations in LUMO energies within it further showcases the richness of structural and electronic diversity achievable with PTCDA. These artificial PTCDA networks hold significant potential for manipulating disordered surfaces, such as metal-intercalated TMDs, and for facilitating the design of novel heterostructures with exotic layers, including metal–organic kagome lattices. The tunable geometric, electronic, and even magnetic properties via choices of metals, molecules, and growth temperatures will extend the functionalities of heterostructures for innovative applications.

## 5 Materials and Methods

### 5.1 Crystal Growth

The single crystals of Fe_0.24_TaS_2_ were prepared by the chemical vapor transport method, using CBr_4_ as the transport agent. High-purity Fe, Ta, and S powders were mixed with a small amount of CBr_4_ (30 mg), sealed in an evacuated quartz tube, and then heated for 10 days in a two-zone furnace with a source zone temperature of 900 °C and a growth zone temperature of 800 °C. Finally, the quartz tube was cooled to 30 °C for 3 days. The obtained single crystals are hexagonal in shape with typical dimensions of 4 × 4 × 0.8 mm^3^. The crystal structure was characterized using powder XRD (Bruker D2 phaser diffractometer) with Cu–Kα radiation. The single crystal quality and crystallization directions were identified by the Laue diffraction method (Photonic Science). The composition ratio of the crystals was acquired using energy-dispersive X-ray spectroscopy (EDS) analysis. Quantitative EDS analysis yields an average atomic ratio of Ta:Fe:S around 4:1:8. From multiple sampling points, the Fe-to-Ta ratio was determined to be 0.244 ± 0.005.

### 5.2 MOF Synthesis and STM/XPS Measurements

The Fe_0.24_TaS_2_ crystals were cleaved under ultrahigh vacuum (below 1 × 10^–9^ mbar) for the acquisition of pristine surfaces. Immediately after cleavage, 40 s of thermal deposition of PTCDA was performed at a rate around 0.015 ML/s and sublimation around 330 °C onto as-cleaved Fe_0.24_TaS_2_ surface at room temperature. This was followed by a postannealing step to modify the structure and properties of the deposited layer. The samples were then transferred to an Omicron low-temperature STM at 78 K under a base pressure of around 2 × 10^–10^ mbar. STS measurements were carried out using the lock-in technique with a 10 mV and 1.3 kHz voltage modulation. X-ray photoelectron spectroscopy (XPS) measurements were performed with monochromatic Al Kα emission (1.4 keV) at room temperature to confirm the formation of the Fe–PTCDA coordination network. The samples were promptly transferred to the XPS system after STM measurements.

## Supplementary Material



## Notes

EDS element ratio analysis and mapping of cleaved Fe_0.24_TaS_2_ crystal; XPS spectra of Fe-PTCDA/Fe_0.2_4TaS_2_ on different energy regions; additional STM dI/dV spectrum taken on herringbone PTCDA area (PDF)

## ∇

Y.-H.C. deceased during revision.
