# Supplementary material for: Temperature-Tunable 2D Assembly of PTCDA on Fe-Intercalated TaS2
Source: ACS Omega. 2025 Jul 22;10(35):39833–40. doi: 10.1021/acsomega.5c03930 (PMC12423968; doi:10.1021/acsomega.5c03930)
Supplement: Supplementary file 1 [file ao5c03930_si_002.pdf]

Temperature-tunable 2D assembly of PTCDA on Fe-intercalated TaS_2

Hou-Ju Chen,[†,#] Yu-Hsun Chu,[†,‡,#] Po-Hsi Huang,[†] Chia-Nung Kuo,[¶,§] Chin Shan Lue,[¶,§] and Minn-Tsong Lin*,[†,∥,⊥]

†*Department of Physics, National Taiwan University, Taipei 10617, Taiwan*

‡*Y.-H. Chu Deceased during revision.*

¶*Department of Physics, National Cheng Kung University, Tainan 70101, Taiwan*

§*Taiwan Consortium of Emergent Crystalline Materials (TCECM), National Science and Technology Council, Taipei 10601, Taiwan*

∥*Institute of Atomic and Molecular Sciences, Academia Sinica, Taipei 10617, Taiwan*

⊥*Research Center for Applied Sciences, Academia Sinica, Taipei 11529, Taiwan*

#*H.-J. Chen and Y.-H. Chu contributed equally to this work.*

E-mail: mtlin@phys.ntu.edu.tw

This Supporting Information outlines the experimental procedures employed in the study and provides three additional figures to complement the main manuscript.

Contents

1. EDS results

2. XPS results

3. Additional STM dI/dV spectra

1. EDS results

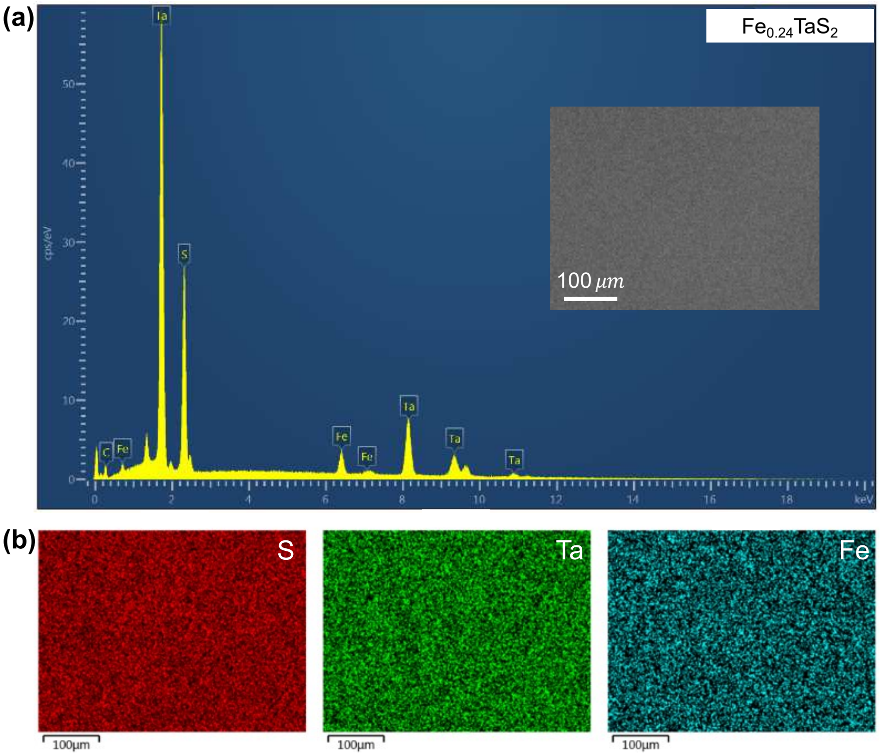

Figure S1: (a) EDS analyzed the element ratio of cleaved Fe_xTaS_2 crystal. (b) EDS mapping for the SEM image (inset in (a)); the corresponding elemental mapping images of the S, Ta, and Fe atoms, respectively.

Supporting Note 1: Energy-dispersive X-ray spectroscopy (EDS) was conducted to analyze the elemental composition and distribution of the cleaved Fe_xTaS_2 single crystals. As shown in Figure S1a, a representative EDS spectrum was acquired from a cleaved surface of the crystal, and the inset displays the corresponding scanning electron microscopy (SEM) image of the analyzed region. The EDS mapping results for sulfur (S), tantalum (Ta), and iron (Fe) are presented in Figure S1b.

The elemental maps reveal that all three elements are homogeneously distributed across the analyzed region, indicating a uniform intercalation of Fe into the layered TaS_2 structure. Quantitative analysis of the EDS data yields an approximate atomic ratio of Ta:Fe:S $\approx$ 4:1:8. The Fe-to-Ta ratio was statistically evaluated from multiple sampling points and determined to be 0.244 ± 0.005.

2. XPS results

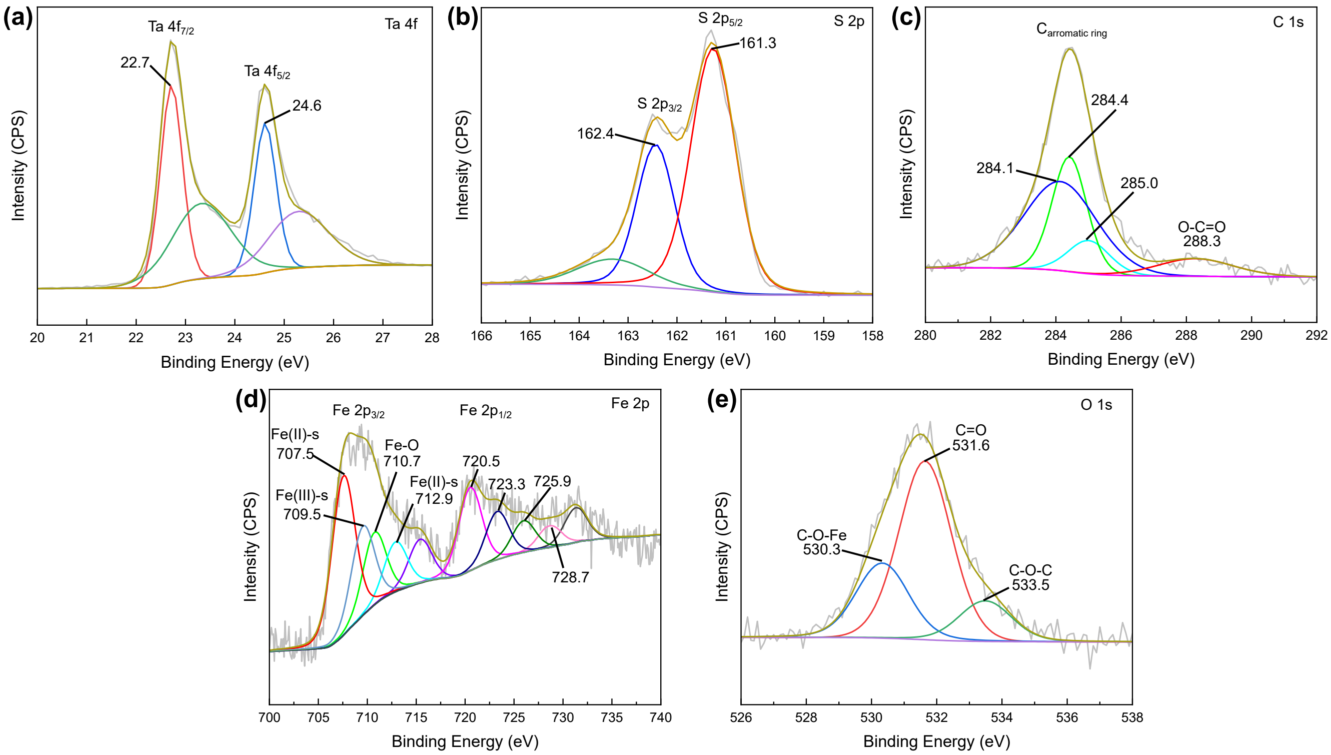

Figure S2: XPS spectra of Fe-PTCDA/$Fe_{0.24}TaS_2$ on different regions. (a)Ta 4f (b)S 2p (c)C 1s (d)Fe 2p and (e)O 1s

Supporting Note 2: X-ray photoelectron spectroscopy (XPS) measurements were performed at room temperature to confirm the formation of the Fe–PTCDA coordination network, as observed in the STM analysis. The TaS_2 substrate was mechanically cleaved under ambient conditions, followed by the thermal deposition of PTCDA for 40 seconds. (∼0.6 ML) Post-deposition annealing was conducted at 150^oC to promote molecular ordering and coordination before the sample was transferred for the XPS measurements.

As shown in Figure S2a, the Ta 4f core level spectrum exhibits two distinct peaks at binding energies of 22.7 eV (Ta $4f_{7/2}$) and 24.6 eV (Ta $4f_{5/2}$), consistent with previously reported values for bulk 2H-TaS_2.[1] In the S 2p region (Figure S2b), the peaks at 161.3 eV (S $2p_{3/2}$) and 162.4 eV (S $2p_{1/2}$) further support the preservation of the underlying TaS_2 sublayer structure.[2]

The C 1s spectrum (Figure S2c) reveals two major components, with the main peak

at 284.4 eV corresponding to the aromatic carbon atoms in the perylene core, and the second component at ~285.7 eV attributed to the anhydride functional groups of PTCDA molecules,[3–5] confirming the presence of PTCDA on the surface.

To gain insight into the electronic environment of Fe in the Fe–PTCDA network, the Fe 2p and O 1s core-level regions were analyzed (Figure S2d). The Fe 2p signal is relatively weak due to the low Fe atomic concentration. The Fe 2p region shows broad features with satellite peaks, suggesting the presence of multiple bonding environments. Peaks at 707.5 eV and 712.9 eV are assigned to Fe^{2+}–S interactions, while the shoulder at 709.5 eV is indicative of Fe^{3+}–S bonding.[6] Notably, the peak at 710.7 eV is attributed to Fe–O coordination, implying interaction between Fe atoms and oxygen atoms from the PTCDA anhydride moieties.

In the O 1s spectrum, three distinct peaks are observed: the peak at 533.5 eV corresponds to the C–O–C bond, while the peak at 531.6 eV is assigned to the carbonyl (C=O) group. A lower binding energy feature at 530.3 eV is attributed to the formation of C–O–Fe bonds, further supporting the coordination interaction between Fe and PTCDA.[6]

3. Additional STM dI/dV spectra

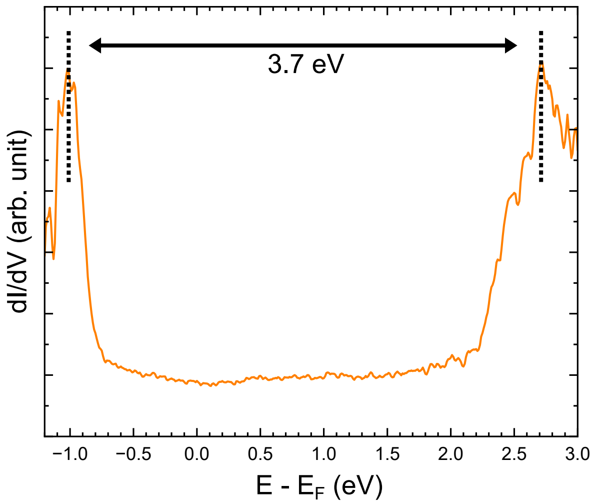

Figure S3: STM dI/dV spectrum taken on herringbone PTCDA area (set point: V_{Tip} = 2.5 V, I_{Tip} = 200 pA)

Supporting Note 3: To investigate the electronic structure of PTCDA molecules assembled in the herringbone configuration on the $Fe_{0.24}TaS_2$ surface, scanning tunneling spectroscopy (dI/dV) measurements were performed over an energy range from –1.2 eV to +3.0 eV. As shown in Figure S3, the differential conductance spectrum acquired on the PTCDA herringbone phase exhibits two distinct features: a pronounced peak at approximately –1.0 eV and another at around +2.7 eV. The uniform LUMO double-lobe found in 2.6 eV in Fig. 2b corresponds the peak at +2.7 eV to the lowest unoccupied molecular orbital (LUMO). The sharp peak found at -1.0 eV can be assigned to HOMO energies and the energy interval between the HOMO and LUMO levels is about 3.7 eV.

References

(1) Zhao, R.; Grisafe, B.; Ghosh, R. K.; Holoviak, S.; Wang, B.; Wang, K.; Briggs, N.; Haque, A.; Datta, S.; Robinson, J. Two-dimensional tantalum disulfide: controlling structure and properties via synthesis. *2D Materials* **2018**, *5*, 025001.

(2) Kovalska, E.; Roy, P. K.; Antonatos, N.; Mazanek, V.; Vesely, M.; Wu, B.; Sofer, Z. Photocatalytic activity of twist-angle stacked 2D TaS2. *npj 2D Materials and Applications* **2021**, *5*, 68.

(3) Schöll, A.; Zou, Y.; Jung, M.; Schmidt, T.; Fink, R.; Umbach, E. Line shapes and satellites in high-resolution x-ray photoelectron spectra of large π-conjugated organic molecules. *The Journal of chemical physics* **2004**, *121*, 10260–10267.

(4) Wang, X.; Zheng, J.; Chen, L.; Li, X.; Cao, C. Studies on structure and spectra characterization of Ni-doped PTCDA films. *Journal of Materials Science: Materials in Electronics* **2017**, *28*, 2037–2043.

(5) Lian, X.; Ma, Z.; Zhang, Z.; Yang, J.; Liu, Y.; Gu, C.; Guo, R.; Wang, Y.; Ye, X.; Sun, S. et al. Alkali metal storage mechanism in organic semiconductor of perylene-3, 4, 9, 10-tetracarboxylicdianhydride. *Applied Surface Science* **2020**, *524*, 146396.

(6) Li, L.; Ma, P.; Hussain, S.; Jia, L.; Lin, D.; Yin, X.; Lin, Y.; Cheng, Z.; Wang, L. FeS 2/carbon hybrids on carbon cloth: a highly efficient and stable counter electrode for dye-sensitized solar cells. *Sustainable Energy & Fuels* **2019**, *3*, 1749–1756.
